# Increased allergen-specific IgG4 levels are linked to suppressed IgE titers when analysed by ISAC but not by immunocap

**DOI:** 10.1186/2045-7022-4-S2-O20

**Published:** 2014-03-17

**Authors:** Hans Jürgen Hoffmann, Johannes Schmid, Peter Adler Würtzen, Ronald Dahl

**Affiliations:** 1 Aarhus University, Department of Clinial Medicine, Aarhus, Denmark; 2 ALK-Abello, Experimental Immunology, Hørsholm, Denmark

## 

Allergen specific immunotherapy (ASIT) is the only disease modifying treatment of allergic diseases. A key element in the protective effect is thought to be induction of a specific IgG4 response leading to a decrease in the IgE-mediated allergic symptoms. The effect of ASIT can be ascertained by provocation or skin prick testing. IgE measurement by ImmunoCAP is a sensitive standard procedure for allergy diagnosis and this has recently been supplemented by a single allergen array (ISAC), which employs significantly lower amounts of allergen and may have different assay dynamics. 24 subjects suffering from allergic rhinitis due to grass pollen allergy were stratified on basis of sensitivity in a basophil activation test, to receive standard subcutaneous immunotherapy (n=18) or to an open control group (n=6). Allergen specific IgE and IgG4 was measured at baseline and when reaching maintenance dose by using both the quantitative ImmunoCAP for Phl p1 and Phl p5 and the chip-based semi-quantitative ISAC method (Phl p 1, 2, 4, 5, 6, 7, 11 and 12). Data are presented as mean and 95% CI. ASIT treatment reduced self-reported symptoms from 10,7 to 5,5 (p=0,0001). ASIT increased allergen specific IgG4 levels, both by ISAC (from 0,3 to 5,0 ISAC specific units (ISU) (∆ 4,7 ISU; 2,7-6,7 ISU; p=0,0001) and ImmunoCAP (from 0,1 to 5,2 mg/l, ∆ 5,1 mg/l; 2,3-7,9 mg/ml; p=0,0006). Specific IgE increased from 23,0 to 48,8 kU/l when measured by ImmunoCAP (∆ 25,8 kU/l; 4,4-47,2; p=0,01). Surprisingly, ISAC measurement of specific IgE decreased from 21,4 to 2,5 ISU (∆ 18,9 ISU; -10,0;-27,8; p=0,0002) during updosing. No significant changes in IgG4 or IgE measurements were found in the control group during the treatment period. ImmunoCAP measurements indicate that IgG4 and IgE increase during ASIT updosing as previously reported. In contrast, when IgE in the same serum samples was measured by ISAC a decrease in IgE seems to be the result of successful ASIT. This may be the result of inhibition of IgE-binding in vitro in the ISAC assay, possibly reflecting functional IgE inhibition in vivo in the treated patients. ISAC measurements before and after ASIT may thus be useful in monitoring the clinical effect of allergen specific immunotherapy.

